# Supplementary material for: Progression of Diabetic Kidney Disease and Gastrointestinal Symptoms in Patients with Type I Diabetes
Source: Biomedicines. 2023 Sep 29;11(10):2679. doi: 10.3390/biomedicines11102679 (PMC10604159; doi:10.3390/biomedicines11102679)
Supplement: Supplementary file 1 [file biomedicines-11-02679-s001.zip › Supplement S1.pdf]

Supplement S1

14. In the last two months, how often have you had pain or discomfort in your stomach?

1. Not at all; go to question 16.
2. Sometimes; less than one quarter of the time
3. Often; more than one quarter of the time
4. Very often; more than half of the time
5. Almost always; more than three quarters of the time

15. In the last two months, how severe has this pain or discomfort in your stomach usually been?

1. Very mild; can usually be ignored
2. Mild; can be ignored if I don't think about it
3. Moderate; cannot be ignored, but does not affect my lifestyle
4. Severe; affects my lifestyle
5. Very Severe; affects my lifestyle a great deal

16. In the last two months, how often have you lost your appetite (did not feel hungry)?

1. Not at all
2. Sometimes; less than one quarter of the time
3. Often; more than one quarter of the time
4. Very often; more than half of the time
5. Almost always; more than three quarters of the time

17. In the last two months, how often have you had heartburn (a burning pain or discomfort that rises up behind the breastbone to your throat)?

1. Not at all
2. Sometimes; less than one quarter of the time
3. Often; more than one quarter of the time
4. Very often; more than half of the time
5. Almost always; more than three quarters of the time

18. In the last two months, how often have you had bitter or sour tasting fluid that comes up into your throat or mouth?

1. Not at all
2. Sometimes; less than one quarter of the time
3. Often; more than one quarter of the time
4. Very often; more than half of the time
5. Almost always; more than three quarters of the time

19. In the last two months, how often have you had a feeling of abdominal fullness or bloating?

1. Not at all
2. Sometimes; less than one quarter of the time
3. Often; more than one quarter of the time
4. Very often; more than half of the time
5. Almost always; more than three quarters of the time

20. In the last two months, how often have you had difficulty swallowing food or liquids (ie. you have noticed food or drink sticking or not passing down properly)?

1. Not at all
2. Sometimes; less than one quarter of the time
3. Often; more than one quarter of the time
4. Very often; more than half of the time
5. Almost always; more than three quarters of the time

21. In the last two months, how often have you felt uncomfortably full soon after starting to eat, so you could not finish your normal meal?

1. Not at all
2. Sometimes; less than one quarter of the time
3. Often; more than one quarter of the time
4. Very often; more than half of the time
5. Almost always; more than three quarters of the time

22. In the last two months, how often have you had an unpleasant feeling of food staying in your stomach after normal meals?

1. Not at all
2. Sometimes; less than one quarter of the time
3. Often; more than one quarter of the time
4. Very often; more than half of the time
5. Almost always; more than three quarters of the time

23. In the last two months, how often have you felt nauseated (you felt like vomiting but didn't)?

1. Not at all
2. Sometimes; less than one quarter of the time
3. Often; more than one quarter of the time
4. Very often; more than half of the time
5. Almost always; more than three quarters of the time

24. In the last two months, how often have you vomited?

1. Not at all
2. Sometimes; less than one quarter of the time
3. Often; more than one quarter of the time
4. Very often; more than half of the time
5. Almost always; more than three quarters of the time

26. In the last two months, how would you describe your usual bowel pattern? *(Tick one answer)*

1. Normal
2. Constipated
3. Diarrhoea
4. Alternating constipation and diarrhoea

If to question no. 26 you answer - Normally, you can not answer the following questions. Thank you for your responsiveness and careful filling of the questionnaire!

27. In the last two months, how often have you had more than three bowel motions a day?

1. Not at all
2. Sometimes; less than one quarter of the time
3. Often; more than one quarter of the time
4. Very often; more than half of the time
5. Almost always; more than three quarters of the time

28. In the last two months, how often have your bowel motions been loose or watery?

1. Not at all
2. Sometimes; less than one quarter of the time
3. Often; more than one quarter of the time
4. Very often; more than half of the time
5. Almost always; more than three quarters of the time

29. In the last two months, how often have you lost control with leakage of bowel movements (accidents)?

1. Never;
2. About once a month or less
3. Two or three times a month
4. About once a week
5. Several times a week
6. Daily

30. In the last two months, how often have you had less than three bowel motions a week?

1. Not at all
2. Sometimes; less than one quarter of the time
3. Often; more than one quarter of the time
4. Very often; more than half of the time
5. Almost always; more than three quarters of the time

31. In the last two months, how often have your bowel motions been very hard or difficult to pass?

1. Not at all
2. Sometimes; less than one quarter of the time
3. Often; more than one quarter of the time
4. Very often; more than half of the time
5. Almost always; more than three quarters of the time

32. In the last two months, how often have you felt that there was still bowel motion that needed to be passed after finishing a bowel motion?

1. Not at all
2. Sometimes; less than one quarter of the time
3. Often; more than one quarter of the time
4. Very often; more than half of the time
5. Almost always; more than three quarters of the time
