# Supplementary material for: Progression of Diabetic Kidney Disease and Gastrointestinal Symptoms in Patients with Type I Diabetes
Source: Biomedicines. 2023 Sep 29;11(10):2679. doi: 10.3390/biomedicines11102679 (PMC10604159; doi:10.3390/biomedicines11102679)
Supplement: Supplementary file 1 [file biomedicines-11-02679-s001.zip › Supplement S2.pdf]

Supplement S2

Preparation before colonoscopy examinations in people with type 1 diabetes

Glycaemic control

- Check your blood glucose level at least every 2-4 hours. If necessary, also at night.
- Frequent monitoring is necessary due to reduced nutritional intake and increased risk of hypoglycaemia.

Hyperglycaemia

- If necessary, correct hyperglycaemia (high blood glucose) using short-acting insulin.
- Due to the risk of hypoglycaemia, correction is recommended only if the glucose level is 10.0 mmol/l and higher:

Glucose level	Short-acting insulin dose for correction of hyperglycaemia
10.0 – 12.0 mmol/l	1 unit
12.1 – 14.0 mmol/l	2 units
14.1 – 16.0 mmol/l	3 units
16.1 – 18.0 mmol/l	4 units
18.1 – 20.0 mmol/l	5 units

Hypoglycaemia

- In the event of hypoglycaemia, which is characterized by a blood glucose level below 4.0 mmol/L, it is recommended to consume a sweetened drink or glucose tablets without delay.
- To minimize the risk of hypoglycaemia, it is advisable to maintain a blood glucose level between 6.0-10.0 mmol/L during the day of the examination, as a significant decrease in food intake can increase the likelihood of this condition.
- In case of hypoglycaemia, it is necessary to take at least 20 g of carbohydrates, for example:
  - 4 teaspoons of sugar
  - 200 ml of sweetened apple juice
  - 5 glucose tablets.
- It's important to keep in mind that beverages with colour, particularly those that are red or purple, should be avoided when preparing for a colonoscopy. Instead, opt for sweetened drinks that are colourless or glucose tablets if you need to manage hypoglycaemia.
- If you have consumed carbohydrates, it's recommended that you check your glucose levels again after 15-20 minutes. If hypoglycaemia persists, consume an additional 20 g of carbohydrates.

- It's important to be aware of the possibility of recurrent hypoglycaemia and to have sugar, sweetened drinks, or glucose tablets on hand and easily accessible.

Diet before colonoscopy

- About 5-7 days before the colonoscopy, it is recommended to eat a light diet with a reduced amount of fibre
  - proteins - steamed or boiled meat/fish, as well as eggs, cottage cheese
  - do not eat red meat
  - slightly unsaturated fatty acids - olive oil, avocado, fatty fish
  - "fast" carbohydrates - white rice, pasta, boiled potatoes without skin, white bread
  - less vegetables – about a quarter of a portion, few fruits (0-1 per day)
  - "fast" carbohydrates and less vegetables are recommended only before colonoscopy, it is not suitable for long-term nutrition of type 1 diabetes patients
- The day before colonoscopy, when you'll be consuming the preparation solution, it's permissible to have a light breakfast and lunch consisting solely of "white" foods. For other meals, opt for beverages that are rich in carbohydrates (such as apple juice and clear lemonades) and proteins (like broths), or snack on sweets such as lollipops and jelly candies. Additionally, you may enjoy sweetened tea and coffee without milk.
- Example of diet:
  - juice jelly dessert (e.g., White)
  - tea or coffee without milk
  - broth
  - lollipops
  - apple juice
- It is not recommended to consume coloured foods/drinks (ready-made bouillon cubes, red, purple foods or drinks) before the colonoscopy.
- Be sure to drink at least 2 L of water to avoid dehydration.
- For diabetic patients, colonoscopy is usually scheduled before 12:00 p.m.

Insulin doses before colonoscopy

- If your blood glucose level is above 5.0 - 6.0 mmol/L, it's important to use short-acting insulin to account for beverages containing carbohydrates and sweets.
- Keep in mind that it may be necessary to decrease the usual dose of short-acting insulin when calculating the amount of carbohydrates in a meal. Consider reducing it by 50%.
- It's crucial to never miss a dose of long-acting insulin.
- You may need to lower the amount of long-acting insulin you take.
- If possible, reduce the dose of long-acting insulin by 20% - 30% in advance, before you start preparing for the examination.
  - you have experienced hypoglycaemia during the night, early in the morning
  - has had hypoglycaemia with long intervals between meals
  - you have managed without short-acting insulin by eating small carbohydrate snacks*

- Make sure to bring your insulin, a glucometer, and either glucose tablets or a sweetened beverage to the examination.

Use of insulin pumps

- Reduce the usual bolus doses before meals by 50%, as well as correct hyperglycaemia (>10 mmol/l) according to the scheme shown above
- Basal input may need to be reduced to 70 – 80% if
    - you have experienced hypoglycaemia during the night, early in the morning
    - has had hypoglycaemia with long intervals between meals
    - you have previously gone without short-acting insulin by eating small snacks
- A reduced basal input should be maintained until the end of the examination
- If in doubt, contact the diabetes education nurse or endocrinologist.
