# Supplementary material for: Progression of Diabetic Kidney Disease and Gastrointestinal Symptoms in Patients with Type I Diabetes
Source: Biomedicines. 2023 Sep 29;11(10):2679. doi: 10.3390/biomedicines11102679 (PMC10604159; doi:10.3390/biomedicines11102679)
Supplement: Supplementary file 1 [file biomedicines-11-02679-s001.zip › Supplement_S3.pdf]

Supplement S3

Table S1. Histopathology findings of upper endoscopy in the study groups.

	**Stable diabetic kidney disease, N=17**	**Progressive diabetic kidney disease, N=4**
Hyperaemic gastropathy	4	0
Hyperaemic duodenopathy	1	0
Oesophageal candidiasis	1	0
Gastric intestinal metaplasia	3	0
Active gastritis	1	1
Chronic atrophic gastritis	1	2

Table S2. Histopathology findings of lower endoscopy in the study groups.

	**Stable diabetic kidney disease, N=17**	**Progressive diabetic kidney disease, N=4**
Polyps	1	1
Diverticulosis	1	0
Erosion of sigmoid colon	1	0
Perianal papilloma	1	0
Eosinophilic infiltration	7	1
Lymphoid follicles/ lymphoid aggregates	6	1
Lymphoplasmacytic infiltration	4	1
Lymphocyte and macrophage infiltration	3	0
Stromal fibrosis	3	0
Mononuclear cell infiltration	2	0
Tubular adenomas	1	1
Active inflammation	5	1
Active colitis	1	0
